# Developing institutional infrastructure for physician wellness: qualitative Insights from VA physicians

**DOI:** 10.1186/s12913-019-4783-9

**Published:** 2020-01-03

**Authors:** Rachel Schwartz, Tait D. Shanafelt, Christophe Gimmler, Lars Osterberg

**Affiliations:** 1 VA Palo Alto Health Care System, Center for Innovation to Implementation (Ci2i), 795 Willow Road, 152-MPD, Menlo Park, CA 94025 USA; 2 Stanford University School of Medicine, 1265 Welch Road, Stanford, CA 94305 USA; 3 WellMD and WellPhd Center, Stanford University School of Medicine, 300 Pasteur Drive, Suite H3215, Stanford, CA 94305 USA; 4 VA Palo Alto Health Care System, 3801 Miranda Ave, Palo Alto, CA 94304 USA

**Keywords:** Qualitative research, Balint group, Burnout, Physician wellness

## Abstract

**Background:**

The prevalence and detrimental effect of physician burnout requires new strategies for supporting physicians. In this project, we describe the development, and assessment, of a “Balint-like” physician support group that provided social cohesion and delivered novel didactic curricula for building resilience.

**Methods:**

The project began with a nine-month facilitated peer-support group for physicians that met every other week. Based on input from the first group, tailored content was developed to address physician wellness needs. These curricula were delivered to participants in the second nine-month Balint-like group. We then conducted semi-structured interviews with 7 hospitalists and 2 outpatient primary care physicians who participated in the Balint-like groups to explore the intervention’s value and to identify remaining unmet physician wellness needs. Using an inductive thematic analysis approach, we identified a set of institutional-, community- and individual-level factors affecting physician wellness and corresponding intervention opportunities.

**Results:**

Physicians spoke of systems-level factors that contributed to distress, and proposed infrastructure, both physical and procedural, that they felt could better support physician wellness. They highlighted the emotional challenges of daily work, and the need for a forum by which to process these interactions in order to maintain their own wellness. Participants reported that participation in Balint-like groups provided this forum and served to help the physicians normalize struggles, reduce isolation and provide new strategies for navigating challenging interactions.

**Conclusions:**

Institutional infrastructure, in the form of regular, psychologically-safe forums for processing with peers and learning relational strategies for preserving wellness, may mitigate physician distress. This project provides a model for how to develop and deliver a low-cost physician wellness program that can be tailored to the needs of individual clinical units.

“Can you form a personal relationship in an impersonal institution?”

—Francis Peabody [1].

National studies have shown that more than half of physicians, across specialties, report experiencing burnout, and the numbers are rising [2]. Burnout, a syndrome primarily afflicting those engaged in people-oriented work, is characterized by emotional exhaustion, increased depersonalization or cynicism, and a sense of inefficacy or lack of personal accomplishment [3]. Physicians’ work-related distress is manifested by increased rates of physician attrition [4, 5], reduced productivity [6] and increased medical errors [7–9]. Provider burnout negatively affects patient care by contributing to a two-fold decrease in patient satisfaction and poorer patient outcomes [10]. On a personal level, the high demands of practicing medicine are associated with physicians’ relationship challenges [11, 12], depression [13] and suicide [14]. In order for physicians to navigate today’s professional demands without damaging their own wellbeing, new strategies are needed to address the factors that detract from professional fulfilment and meaningful engagement. A broad array of strategies have been recommended to improve physician well-being, each of which can be accomplished with a variety of specific tactics [15–18] . To date, few of these strategies have been systematically implemented in primary care or hospital medicine.

## Background

In detailing the factors necessary to restore joy in practice, Swensen & Shanafelt [17] stress that organizations need to provide physicians with the necessary infrastructure to support meaningful engagement. High work hours, productivity expectations, clerical burdens and administrative demands curtail the ability of physicians to connect with colleagues and contribute to isolation. The time pressure physicians are under also constrains meaningful engagement with patients; a study by Detmar and colleagues showed that when patients’ appointments started later than the scheduled time, patients’ emotional problems were significantly less likely to be discussed [19]. Patients also detect physician distress, using cues that include work pace, physician demeanor, and physical appearance, and these perceptions impact the doctor-patient relationship [20]. Physicians report that their relationships with patients contribute to the greatest portion of their job satisfaction [21–23], which suggests new strategies are needed to facilitate engagement that will be rewarding for provider and patient alike.

Providing opportunities for physicians to connect with colleagues has been shown to positively impact professional satisfaction [24], imparting to physicians a sense of security and tools for “emotionally metabolizing” the more difficult aspects of their job [25]. Community and collegiality-building practices for health care providers are known to be beneficial for improving wellbeing and reducing burnout [16, 26, 27]. There are multiple models for implementing supportive groups for care providers, ranging from the Mayo COMPASS groups or dinner groups [27, 28], to Balint groups [24] and Schwartz rounds [29], to name a few. There is growing evidence that a group process approach has yielded benefits across a variety of practice settings [24, 30–32].

In this project, we explored the factors that contribute to physician wellness and the challenges and joys of their work life, and evaluated the effect that participating in the Balint-like group had on physicians’ well-being. Balint groups are regular meetings of 6–12 physicians, facilitated by one or two trained leaders [25]. The group begins with a clinical case presentation, followed by a discussion that builds from the case and may cover a range of themes, from challenging patient interactions, to work-life balance, to system barriers, communication strategies, and personal issues [33, 34]. While the traditional Balint group is facilitated by a psychoanalyst and a physician, without didactic content [35], our Balint-like group, similar to that of Rabin and colleagues [33], included curricular content designed to provide participating physicians with skills to deal with physician-patient and workplace challenges. The project’s objective was to qualitatively examine the effects of a 9-month long Balint-like intervention on VA hospitalist and primary care physicians’ well-being and to identify remaining unmet needs.

## Methods

### Methodology/research design

#### Project overview

This project consisted of two consecutive 9-month Balint-like groups, the first without curriculum and the second integrating didactic content that was developed in response to feedback from participants in the first group. Finally, we conducted individual qualitative interviews with participants from both Balint-like groups to understand the effect of group participation and to identify additional barriers to physician wellness.

### Balint-like groups

We conducted Balint-like groups with VA hospitalists and primary care physicians over the course of two consecutive nine-month periods. Participants were recruited through announcements at staff meetings, through staff email lists, and by word-of-mouth. The first nine-month long Balint-like group consisted of general medicine outpatient doctors and did not include curricular content, only the case presentation structure. The second nine-month long Balint-like group consisted entirely of hospitalists and included didactic content in addition to the standard group process. Please see Table 1 for a description of the curricular content.

**Table 1:** Curricular module content

Module	Central Conceptual Framework	Relevant Capacity/Skill	Practical Application To Practice
Strengthening Emotional Balance	Sequenced stages of emotional episode: Trigger, Emotion Program, and Emotional Behavior	1) Understand and analyze personal conditioning & emotional triggers 2) Feel sensate/somatic experience of emotions directly 3) Differentiate conscious from unconscious emotional behavior	Building capacity to consciously observe, experience, and act from emotions constructively rather than destructively
Tracking and Regulating Stress Responses	Mapping angry and anxious activation vs. overwhelmed shutdown in self & other	1) Develop interoceptive skills: monitoring internal stimuli 2) Regulate stressful responses and energize shutdown states	Enhancing ability to sense activation or shutdown & then intervene to self-regulate
Interpersonal Boundaries	1) Function of healthy boundaries 2) Spectrum and features of loose and rigid boundaries	1) Identify own typical boundary style 2) Verbalize personally appropriate boundaries	Maintain relational safety amidst challenging interactions
Priorities in Interpersonal Conflict	Prioritizing between three central priorities and approaches in relational conflict	1) Asking for change in another 2) Preserving relationship 3) Preserving personal boundaries	Effectively managing conflicts or disagreements
Managing Disruptive Patients	1) Assessing levels of stress in patients 2) Using the appropriate approach per stress level	1) Non-verbal de-escalation 2) Active and reflective listening 2) Limit-setting	Developing confidence in assessing and responding to disruptive patients

The first Balint-like group of practicing physicians in primary care was launched in 2016, and feedback was gathered from participants that was used to further hone the curricular content before it was delivered as part of the 2017 Balint-like group. One of the authors (CG) an internist and Marriage and Family Therapist (specializing in health care provider clientele), was responsible for developing and delivering the curricula, which provided physicians with new psychological strategies and interpersonal tools to use in a clinical setting. Balint sessions were 75 min each, held every 2 weeks over lunch for a 9-month period.

### Data collection: evaluation of Balint-like groups

Formal individual interviews were conducted with nine Balint group participants after the completion of the nine-month Balint-like group sessions to qualitatively evaluate impact. The study was reviewed by the Stanford University Institutional Review Board and approved as a Quality Improvement project. Consent to participate was given verbally in order to further protect the anonymity of all participants.

All interviews were conducted by the first author (RS), a health services researcher with qualitative expertise who had not met any of the participants prior to the interviews. The interviews took approximately 30 min, and were conducted in-person using a semi-structured interview guide (please see Additional file 1). All interviews were audio recorded.

### Data analysis

Audio recorded interviews were deidentified and transcribed. Interview excerpts were then organized by the first author according to interview question, to further preserve participant anonymity since other authors knew the participants. To ensure the trustworthiness of our results, the coding team (RS, CG, LO) independently coded each transcript before meeting to collectively analyze themes using an inductive thematic analysis approach [36]. The approach entailed iteratively grouping codes into themes, identifying overarching themes and associated theoretical concepts. All discrepancies were resolved by consensus. The diversity of our professional backgrounds (one health services researcher and communication scientist, one psychotherapist and internist, one medical educator and internist) further reduced bias by integrating different perspectives. Data saturation was achieved by the end of the nine interviews.

## Results

### Balint-like group participant demographics

Participants included all seven hospitalists from the second Balint-like group (six female, one male), and two general internal medicine physicians (both female) from the first Balint-like group. Only two physicians were selected from the first group to participate because over a year had elapsed since the first Balint-like group had taken place, and there were concerns about the extent to which participants in the first group would be able to remember details from their participation. The two individuals from the first group who participated were selected based on their role as outspoken leaders in the first group who were responsible for shaping the curricular content that resulted. The physicians had an average of 12 years in practice, with a range of 3–20 years (*SD* = 6.40). Physicians from a variety of racial and ethnic backgrounds (Non-Hispanic White, East Asian, South Asian, African American) were represented.

Five content areas were created and designed to be delivered within the context of Balint-like groups when themes emerged in discussion that could be usefully supported by didactic content. Please see Table 1 for a description of the five curricular modules.

### Perception of impact of Balint-like groups on distress

#### Decreased Isolation & Normalizing Struggle

Participants highlighted how the group meetings served to normalize the emotional challenges of practicing medicine and provided a sense of community that had been missing in their professional life:

“I think the most helpful thing for me for is that it was de-stigmatizing … I think that for me, the Balint group had a redemptive quality, because I just heard the other doctors-- it does feel confessional a little bit, the sharing-sharing-- that they have the same thought process. And then I just felt so much tension leave me, like I don’t have to judge myself for feeling this way. I’m not alone, everybody else feels the same, there’s certain patients that trigger them or they want to avoid. So that for me was absolutely the best part of the Balint group is that I really don’t judge myself as much, because I’m like, I think I’m pretty normal. [JA11].

Participants spoke to how the group process changed the culture on the team, providing new opportunities for more personal engagement:

“I think one major thing was just hearing about ... knowing that other people in our group were also experiencing very similar types of difficult interactions and had similar feelings or trouble coping with certain situations. So that in itself is just helpful. It kind of opens the door, I think, to talk a little bit more with each other about those things and be supportive to each other.” [HE11].

### Opportunities to learn new practices

Multiple participants described the value in learning how their colleagues handled challenging interactions as a way to develop new practices:

“As attendings, we practice pretty independently. I mean, we have our teams, our residents and med students and stuff, but we practice alone. We don’t have a lot of opportunity to observe each other, so a lot of the learning and, yes, you’re still learning a lot as you’re attending each year, is kind of on your own. So it’s really helpful, I think, to hear about how other people approach things because that might give you a different perspective, or different idea, or something else to try out. [HE11]”.

Another example:

“Definitely [the group has] helped me feel more calm at work, so in that sense I’m sure it’s been helpful. It’s on my mind a lot more. When I have an encounter with a patient, I’ll think, “This sounds like something that happened in the group, and this is how this person would’ve handled it.” [SG12].

### Emergent themes regarding unmet physician needs

**Fig. 1:**
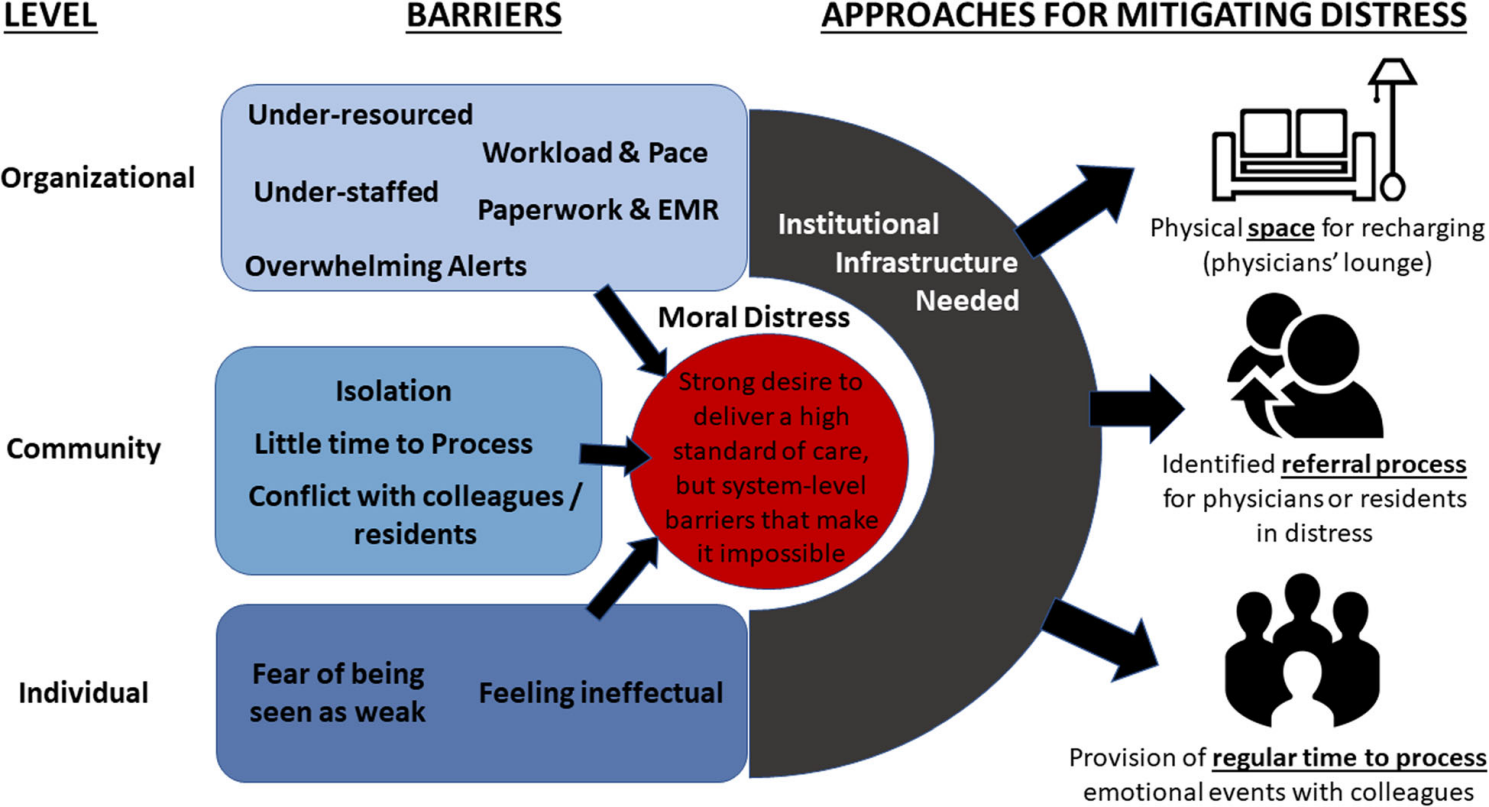
Themes addressing organizational, community, and individual-level physician wellness factors

Unmet needs relating to physician wellness patterned into organizational, community- and individual-level themes, detailed below. Institutional infrastructure, in the form of dedicated space, such as a physicians’ lounge for self-care, and an identified, internally supported dedicated time for physician processing, was highlighted as an unmet need. The disconnect between individuals’ desire to deliver care that met their own standards and their inability to deliver such care due to system-level barriers was a frequent source of distress, frustration, and demoralization. Please see Fig. 1 for an overview of themes and potential approaches for addressing the challenges described.

### Organizational

Organizational themes included challenges navigating a pressured work pace and a workload perceived to be unmanageable, inadequate staffing, large administrative/clerical burden, and inadequate resources to deliver care that adhered to physicians’ own standards.

#### Workload and pace

Physicians repeatedly highlighted the time pressure and an overwhelming volume of work as a core feature of their daily experience. As one person explained:

“The other hard part of the job can be the pace of it. Sometimes you feel so scatter- brained...things happen in the hospital and everything feels so urgent. This person’s calling you and it has to be done right now. But you can imagine sometimes three of these things happen concurrently. It just feels like you can be wound so tight because of it.” [JA11].

Other stress-inducing factors included system-level alerts that require attention and active management. One physician described the experience:

“We get so many alerts on our screens that it can be completely overwhelming. And if you get behind, I think [you] kind of have this sense of like, “Well, that’s it. I can’t catch up, there’s too much to do.“ And it feels overwhelming because you know you have all this work that’s accumulated.” [PI09].

Individual strategies for managing the administrative aspects of medicine varied. Several reported making a conscious decision to prioritize time with patients at the cost of keeping up with administrative/clerical duties:

“I’m negligent on all my documentation and the emails and meetings and all that stuff. I don’t blow it off, but I basically try to get away with as little as possible of that because I’d rather be with the patients.” [SG12].

Another echoed this experience, highlighting the emotional repercussions of this decision:

“I am not good with timely documentation. I don’t do my notes on time because somewhere along the way I made the decision, I’m going to spend my time talking to people instead of writing notes. But to be honest, it’s not okay from a facility level. Doctors have to document and they have to document in a timely manner. So I’ve really cut corners in that way, and I feel shame about it and I know that other people probably struggle with it too, but I know that other people also get their documentation done faster than I do.” [JA11].

### Community

Multiple participants described feeling isolated at work. This was in part attributed to the high work volume interfering with time for personal engagement, and in part due to the lack of a forum for having a more personal connection with peers:

“I think it’s very easy to feel alone here. Not that there’s not backup or help or whatever, but I just don’t really sit and talk to anybody.” [PI09].

Another explained:

“Here, we’re all in our own silos. It’s just a wave of the hand and we keep moving. So that’s why I felt like the Balint group helped to bring some of that camaraderie in.” [BA07].

### Need for processing emotional dimensions of work

Physicians discussed the emotional challenges of daily work, and the need to be able to process the interactions in order to not carry the weight home with them. The lack of an institutional protocol or forum for processing for some physicians came at the expense of their families’ and their own emotional wellbeing:

“I honestly don’t feel like I have time to process the majority of the day... I think there’s a true lack of understanding about how much baggage everybody takes home. I think there’s maybe assumptions of us having abilities to kind of turn off or turn on certain things and I don’t think that’s sort of human nature.... we’re not great necessarily at asking for help over that... just the processing of difficult events or patient deaths or unexplained outcomes or ... there’s a lot of stuff that you can take home … I feel like we don’t get much support in that arena and ...that just doesn’t go away … I can tell because I go home and I’m short with my kids or ... other people sort of pay the consequences of it. Or you just sort of learn to keep it to yourself, you know?” [GE02].

### Individual level

Multiple physicians, each at different stages in their career, spoke about their fears of appearing incompetent or weak before colleagues, and how this fear contributed to the anxiety they experienced at work:

“You make yourself vulnerable when you are in a situation where you feel like you cannot solve a problem or fix a problem. And I don’t want my nurse to see me in that vulnerable state.” [BA07].

Another described worrying about how much to disclose during the Balint-like group meetings:

“Sometimes I would share something and it would make me think, “Should I have shared that? These are my colleagues, what if they don’t have the same feelings? What if they think I’m crazy or weak,” or whatever. And the thought comes up.” [SG12].

This fear of inadequacy was compounded by working in a high-stress environment:

“I recognized in myself that I had a high level of anxiety when I was at work, and partly [it’s] anxiety born out of the environment [of constant] emergency, emergency, crisis all the time, but also anxiety about feeling like, am I good at this, am I good enough? Am I good enough to teach other people? Don’t you have to be extra good to do that?” [JA11].

### Moral distress

Moral distress manifest itself in the divide between the quality of care physicians felt committed to delivering and the reality of what they were able to provide. They described feeling ineffectual at being able to relieve patients’ suffering as a result of system-level constraints, and care-team limitations that contribute to frustration:

“One challenging part of the job is knowing what is ideal to take care of the patient and sometimes not feeling like you can achieve that, either not having the right resources or enough resources, or not everybody is working ideally together on the same page in an interdisciplinary manner.” [HE11].

One person described the ethical challenges involved in determining who receives care:

“As a doctor, we are taught to respond to the individual patient’s need without judgment and to weigh their perception of need against our interpretation of their disease process that requires them to actually be in the door talking with us. Often, we don’t see those patients because, as a facility, we have to make important decisions about how do we prioritize need. The very fact that we have to talk about prioritizing need is an ethical dilemma … you’re taught to practice a certain way with the assumption that the system should allow you to practice in that way and often we have limitations that are not in our control and we have to be the bearer of bad news that we can’t provide this service for you because we’re not set up to do so.” [JL02].

### Institutional infrastructure

Physicians spoke to infrastructure, both physical and procedural, that they felt was needed to better support physician wellness. Multiple participants cited a lack of physical space for physicians to attend to their own basic needs:

“The greatest unmet needs are the actually physical and psychosocial emotional space in the workspace to recharge. There is no place to sit down and take a breath. Take a drink of water, have a cup of tea, close your eyes … We don’t have that, and I think we all fantasize about having some space like that.” [SG12].

Adequate staffing support was also highlighted as a factor that contributed to frustration and increased stress:

“We’re seemingly always short staffed on social workers. Our pharmacists have been short staffed … Trying to make the best of what we have can sometimes be challenging. Especially if you don’t have the adequate staff... over the last year or so, it’s been very fragmented, so a lot of things get lost in translation. A lot of rework is done, there’s a lot of misunderstandings and miscommunications.” [HE11].

Finally, physicians noted that there was no established infrastructure to support physicians who may be experiencing burnout. While several spoke to how the Balint-like group built community and lowered the activation energy necessary to discuss professional and personal challenges, they acknowledged that outside of the group, there was no formal support system or identified referral pathway for those in distress.

## Discussion

This project explored unmet needs of Veterans Affairs primary care physicians and hospitalists, and the effect that a Balint-like peer support intervention had in supporting physicians and helping them develop new approaches for navigating professional challenges. The curricula integrated psychological tools and training with communication strategies, leveraging guidance from peers to provide physicians with new tools for navigating professional challenges. Our results both affirm the benefits observed in other Balint groups, including increased confidence and perception of competency at work [24], and improved psychological skills for navigating medicine [37], as well as provide new insights on the sources of physician distress and potential mechanisms by which to effect change.

One of the recurrent themes that emerged from the structured interviews was the relationship between resource constraints and physicians’ frustration and distress. Such distress is consistent with the definition of “moral distress” that has been reported more broadly in health professionals. Moral distress has been defined as the feeling experienced by a health professional when he or she knows the appropriate course of action but is prevented from doing so due to existing obstacles [38, 39]. Institutions should be aware that inadequate resources or other obstacles that constrain care delivery can cause distress and contribute to burnout when physicians feel unable to deliver care that meets their internal standards for what is appropriate. Moral distress has been correlated with burnout and intention to leave practice [40] but proposals for how to mitigate this distress are lacking.

While we did not use quantitative measures, our qualitative data suggest that Balint-like groups may provide one mechanism for alleviating isolation, moral distress and other challenges by normalizing and validating physician’s experiences, creating community and providing insights into how colleagues navigate these challenges. Adding didactic content to Balint groups, tailored to the needs of each institution or unit, that bridges psychological and medical realms may help to further address physicians’ distress by providing new tools for addressing the challenges of clinical practice. Balint-like groups provide a low-cost support mechanism that benefit both individual physicians and the institution through creating community and providing opportunities to learn new practice strategies that can lead to more meaningful clinical encounters. Additional institution-level attention to providing support for physician self-care, in the form of break rooms that enable basic self-care and an established protocol and referral process for physicians in distress, could serve to better address unmet physician needs.

### Limitations

This project served as foundational work to demonstrate opportunities to address physicians’ unmet needs. However, the current study’s sample size was limited. After developing the curriculum and performing this initial evaluation, the impact of this intervention is now ready to be tested with quantitative measures using a larger group of participants. There was representation from two groups of physicians, outpatient primary care physicians and hospitalists, however there were only two outpatient representatives in our qualitative analysis which limits the extent to which the views presented here can be extrapolated. Future studies that specifically focus on the different needs of physicians who work defined shifts versus those who practice in outpatient settings may provide insight on the extent to which work hours and practice environment may differentially affect physician wellness. This study took place at a VA hospital, and VA physicians experience different system-level challenges than other practice environments, which constrains generalizability of findings.

### Conclusions

This project began with a peer-support Balint-like group, followed by curriculum development, based on input from the first group, to provide physicians with new psychological and communication tools for navigating professional demands. This novel curricular support intervention was then delivered through a Balint-like group in an institutionally-supported context. Finally, we conducted a qualitative evaluation of the value of the intervention and remaining unmet needs. Taken together, our results highlight numerous sources of physician distress that can be mitigated through targeted solutions. The importance of providing physicians with a forum for community and opportunities for emotional processing of distressing events cannot be overstated, and the use of Balint-like groups are one effective solution that can be added to the growing list of solutions for addressing physician loneliness and isolation [41, 42]. It would be beneficial for future studies to explore how this intervention should be adapted to best serve a variety of physicians in diverse clinical environments.

## Supplementary information

**Additional file 1.** Interview Guide, Description of data: Interview questions.

## Data Availability

We have included the interview guide in Additional file 1. Individual interview transcripts are not available to preserve the anonymity of participants.

## Abbreviation

VA: Veterans Affairs
