# Supplementary material for: Developing institutional infrastructure for physician wellness: qualitative Insights from VA physicians
Source: BMC Health Serv Res. 2020 Jan 3;20:7. doi: 10.1186/s12913-019-4783-9 (PMC6942336; doi:10.1186/s12913-019-4783-9)
Supplement: Supplementary file 1 — Additional file 1. Interview Guide, Description of data: Interview questions. [file 12913_2019_4783_MOESM1_ESM.docx]

Additional file 1

Interview Guide

Your name will never be associated with the interview. If at any time during the interview you want to stop, we can stop. If at any time after the interview you don’t wish your interview to be included, we can also remove it. The main purpose of this interview is to reflect on the sessions this year and improve them for next year.

1. How old were you when you started to think about becoming a doctor?

a. Was there a specific event or something that led to it?

b. Did that same motivation keep you going all the way through?

c. How did you choose your specialty?

d. Can you describe what you see is your role as a physician?

2. What gives you the most satisfaction in your work?

2a. What’s the most meaningful part?

2b. What percentage of your work time feels meaningful?

3. What are the most challenging parts of your job?

4. What types of interactions at work do you feel like you don’t manage or process as well as you’d like to? How do these types of interactions affect you?

6. If you’d known as a pre-med student what the reality of practicing medicine was like, would you still have chosen this path/specialty, or what decisions would you have made differently?

7. Do you feel the practice of medicine has changed over the course of your career? [if yes: How so?]

8. What advice do you give to med students and residents about how to navigate the challenges of practicing medicine? Do conversations about wellness come up? [if yes, How/when? If not, why not? Time? Power dynamics? Etc.]

10. What aspects of the Balint group were most valuable to you?

11. If you could’ve changed one aspect of the Balint group, what would it be? [prompt: anything else that you’d change about the content/delivery/group discussion?]

12. Of the curricular topics that were covered, which ones do you wish you’d spent more time discussing?

13. What other topics would you like to see covered that weren’t touched on?

14. Were there barriers to sharing as freely as you’d have liked to? [prompts: concerns about how confidential the group was, or other group dynamics that affected your experience?]

15. Has your involvement in the Balint group changed how you approach medicine in any way (modeling, teaching, caring for patients)?

15. What do you feel are the greatest unmet needs relating to physician wellness? What types of solutions do you feel are needed to better meet these needs?

16. Any other questions you wished I’d asked that we haven’t talked about?
